# The outcomes of stand alone polyetheretherketone cages in anterior cervical discectomy and fusion

**DOI:** 10.1007/s00264-020-04760-1

**Published:** 2020-08-16

**Authors:** Abdulaziz F. Ahmed, Mohammed Al Ateeq Al Dosari, Abdulaziz Al Kuwari, Nasser Mehrab Khan

**Affiliations:** 1 Department of Orthopaedic Surgery, Hamad Medical Corporation, PO Box 3050, Doha, Qatar; 2 Aspetar Qatar Orthopaedic and Sports Medicine Hospital, Doha, Qatar

**Keywords:** Polyetheretherketone, PEEK, Cages, Anterior, Cervical, Discectomy, Fusion

## Abstract

The procedure of anterior cervical discectomy and fusion is considered as the treatment of choice in degenerative disc disease, which material provides the best clinical and radiological fusion and other outcomes remains heavily debated. Materials that augment the process of fusion consist of bone grafting, titanium, polyetheretherketone (PEEK), or carbon cages. The application of PEEK cages has been recommended as it is radiolucent, and it has a modulus of elasticity that is similar to cortical bone. PEEK cages can be either filled with various materials or unfilled cages. Filled PEEK cages can include bone autografts, bone allografts, demineralized bone matrix, and other materials that facilitate fusion. This narrative review highlights that standalone filled PEEK cages were likely to have better radiological outcomes and satisfactory clinical outcomes for myelopathy when compared with standalone unfilled PEEK cages.

## Introduction

Neck pain among the population is a prevalent complaint with an incidence of neck pain found to be as high as 67% [1, 2]. Several associated factors have been found to be related with neck issues such history of recurrent headaches, low back pain, and prior trauma [1, 3, 4]. Neck pain as a result of cervical degenerative changes can be due to spinal canal stenosis, facet joint arthropathy, and spondylolisthesis. Such conditions can lead to neck pain that is recalcitrant to conservative treatment and therefore requires surgical intervention. The surgical treatment of cervical degenerative disc disease (DDD) is by decompressing and stabilizing the affected cervical segment through either an anterior or a posterior approach. The anterior approach consists of anterior cervical discectomy and fusion (ACDF) or disc arthroplasty; whereas, the posterior approach can include laminectomy and fusion or laminoplasty.

The procedure of ACDF is considered as the treatment of choice for DDD [5–7]. It encompasses decompression of impinging structures within the cervical spine is followed by stabilization of the decompressed level with plates for fusion. A study of 125 patients who underwent ACDF followed for a mean of 11 years indicated a 96% favourable outcome with only five patients undergoing adjacent level surgery [8]. However, till date, it is heavily debated which material provides the best clinical and radiological fusion in ACDF. Instrumentation for fusion can include the use of titanium, polyetheretherketone (PEEK), carbon cages, or allograft. In addition, the fusion through these instruments can be augmented with the use of bone graft. On the other hand, fusion alone can be performed through solely using bone autograft or allograft without any of the aforementioned instrumentations as well.

PEEK cages have been developed in 1990s which allows performing ACDF with decreased subsidence rates due to its low elastic modulus and due to being radiolucent [9]. PEEK cages can be either unfilled cages or filled with various materials. Filled PEEK cages can include bone autografts, bone allografts, demineralized bone matrix, and other materials that facilitate fusion [10]. Moreover, PEEK cages can be used standalone or can be augmented with additional plate fixation [11]. In this review, we aimed to summarize the latest evidence on the radiological and clinical outcomes following ACDF with standalone PEEK cages for DDD.

## The outcomes of PEEK cages

**Fig. 1:**
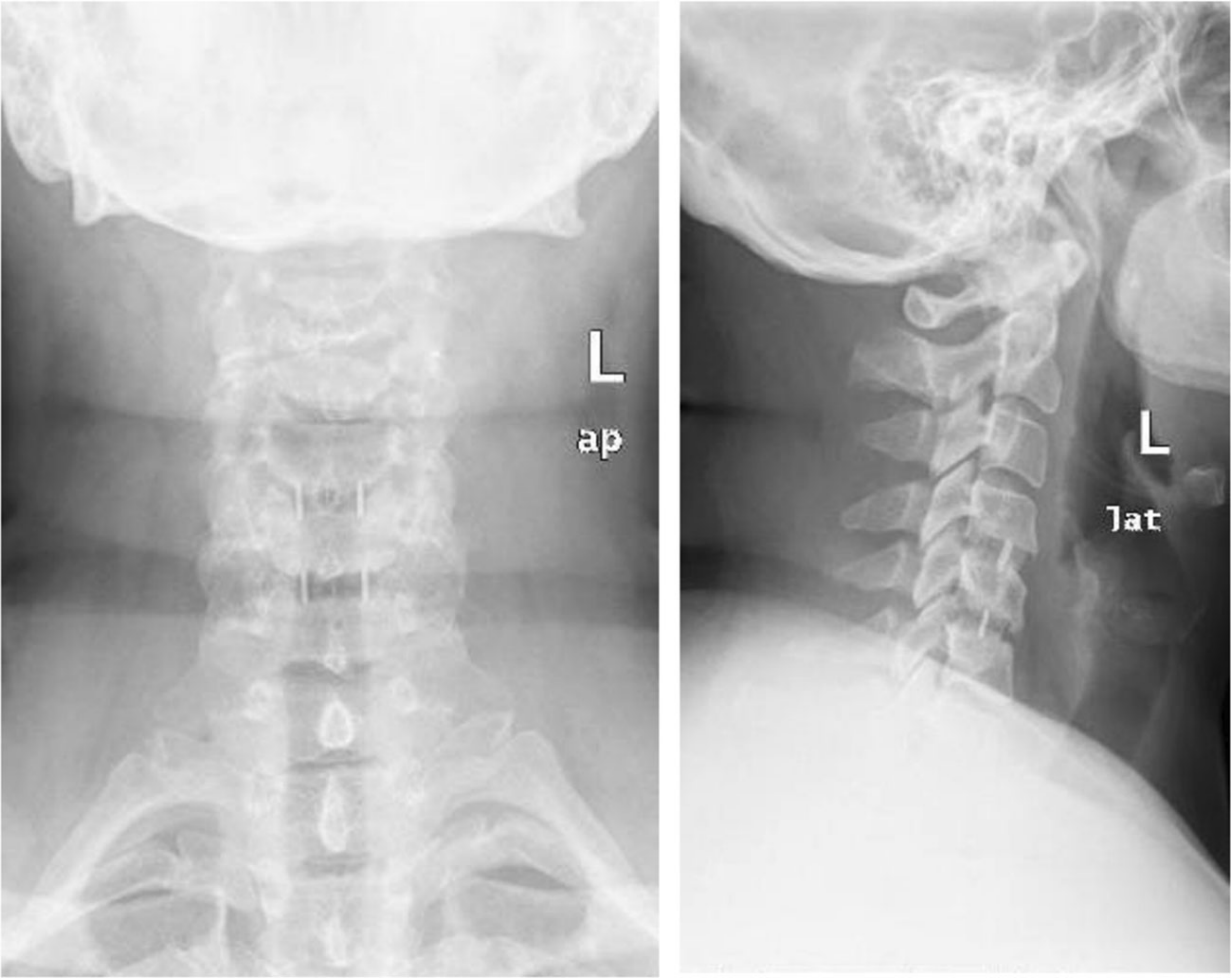
Post-operative radiographs of a 46-year-old female who underwent anterior cervical discectomy and fusion with PEEK cages filled with bone marrow aspirate. Surgical indication was right arm radiculopathy that failed non-surgical treatment

Figure 1 displays the postACDF radiographs with filled PEEK cages. Several studies have looked into PEEK cages in term of clinical and radiological outcomes following ACDF for DDD. Table 1 summarizes the studies searched in our narrative review.

**Table 1:** Characteristics of different studies search in this review

Author, year	Study design	Surgical indications	Patients	PEEK cage type	Mean follow-up
Cho et al., 2002	Prospective CS	Radiculopathy Myelopathy Radiculomyelopathy	22 one-level 10 two-level 8 three-level	Filled with autograft	6 months
Liao et al., 2008	Case Series	Radiculopathy Myelopathy Radiculomyelopathy	19 Number of levels not specified.	Filled with allograft	12 to 18 months (mean not reported)
Lied et al., 2010	Prospective CS	Radiculopathy Myelopathy Radiculomyelopathy No radiculopathy or myelopathy	77 Number of levels not specific per study group.	Empty	6 months
Niu et al., 2010	RCT	Radiculopathy Myelopathy Radiculomyelopathy	16 one-level 9 two-level	Filled with allograft	12 months
Zhou et al., 2011	Prospective CS	Radiculopathy Myelopathy	30 one-level 17 two-level	Filled with autograft	12 months
Cabraja et al., 2012	Retrospective CS	Radiculopathy Myelopathy	42 one-level	Empty	28.4 months
Klingler et al., 2014	Retrospective CS	Radiculopathy Myelopathy	27 one-level 12 two-level	Empty	16 months
Shiban et al., 2015	Case series	Radiculopathy Myelopathy	127 one-level 125 two-level 13 three-level	Empty	21 months
Gok et al., 2016	Case series	Radiculopathy	25 one-level	Empty	16 months
Park et al., 2016	Case series	Radiculopathy Myelopathy Radiculomyelopathy	77 one-level	Empty	21 months for subsidence patients 16 months for non-subsidence patients
Farrokhi et al., 2017	RCT	Radiculopathy Myelopathy Radiculomyelopathy	32 one-level	Filled with bone substitute	12 months
Ofluoglu et al., 2017	Case series	Radiculopathy Myelopathy	16, no level specified	Filled with with ß-TCP	13 months
Suess et al., 2017	RCT	Radiculopathy Myelopathy	239 one-level	Empty	18 months
Kapetanakis et al., 2018	Case series	Radiculopathy	36 one-level	Empty	12 months
Godlewski et al, 2018	Case series	Non-specific	100 one- or multi-level	Filled with phosphocalcic hydroxyapatite	12 months
Kim et al., 2018	Case series	Radiculopathy Myelopathy	60 two-level 8 three-level	Empty	27.6 months

*CS* cohort study, *RCT* randomized controlled trial, *PEEK* polyetheretherketone, *ß-TCP* ß tricalcium phosphate

### Fusion and subsidence rates

Table 2 summarizes the fusion and subsidence rates in our literature review. The definitions of fusion and subsidence rates were variables among studies. Post-operative fusion was defined as the lack of motion in lateral cervical flexion and extension radiographs in three studies [13, 16, 25]. One study defined fusion as the occurrence of anterior and posterior bone bridges on radiographs [21]. Park et al. [24], Farrokhi et al. [12], and Kapetanakis et al. [22] defined fusion by the presence of motion and bone bridging on radiographs. Suess et al. [18] defined fusion with three parameters consisting of bony bridging, absence of radiolucency at the implant-vertebrae interface, and the lack of motion on dynamic x-rays. Cho et al. [14] utilized radiographs to assess fusion without any further details on how fusion was objectively measured. Three studies determined fusion through evaluating motion on lateral cervical radiographs and bone bridging on computed tomography (CT) scan [20, 23, 30]. Zhou et al. [15], Liao et al. [16], and Klingler et al. [19] evaluated fusion by using CT scans only. The subsidence rate was evaluated vaguely by radiographs in three studies [14, 16, 23]. Three studies defined subsidence as the radiographic change of at least 3 mm in the interbody space [13, 17, 24, 25], whereas another two studies considered subsidence as a change of at least 2 mm in the interbody distance [21, 22]. Kim et al. [20] defined subsidence as a decrease of interbody height by more than 2.5 mm on lateral radiographs. Klingler et al. [19] measured subsidence by comparing pre- and post-operative total segmental heights without a predefined threshold for subsidence.

**Table 2:** Summary of fusion and subsidence rates in different studies on standalone PEEK cages

Author, year	Type of PEEK cage	Fusion rate	Subsidence rate	Mean follow-up
Cho et al., 2002	Filled with autograft	100%	0%	6 months
Liao et al., 2008	Filled with allograft	100%	0%	Range: 12–18 months
Niu et al., 2010	Filled with allograft	100%	0%	12 months
Zhou et al., 2011	Filled with autograft	100%	-	12 months
Cabraja et al., 2012	Empty PEEK cages	88.1%	14.3%	28.4 months
Klingler et al., 2014	Empty PEEK cages	65%	31%	16 months
Shiban et al., 2015	Empty PEEK cages	85% in one-level 95% in two-levels 94% in three-levels	25% in one-level 27% in two-levels 15% in three-levels	21 months
Gok et al., 2016	Empty PEEK cages	92.3%	33.8%	16 months
Park et al., 2016	Empty PEEK cages	92%	12%	12 months
Farrokhi et al., 2017	Filled with bone substitute	93.8%	-	12 months
Kapetanakis et al., 2017	Empty PEEK cages	100%	0%	12 months
Ofluoglu et al., 2017	Filled with with ß-TCP	95%	-	13 months
Suess et al., 2017	Empty PEEK cages	83%	-	18 months
Godlewski et al, 2018	Filled with phosphocalcic hydroxyapatite	-	10.23	12 months
Kim et al., 2018	Empty PEEK cages	81.3%	48.3%	27.6 months

*PEEK* polyetheretherketone

In terms of filled PEEK cages, the reported rate of fusion and subsidence varied between 94.3–100% and 0–10%, respectively in our literature review. Farrokhi et al. [12] reported 93.8% fusion rate at 12 months in an RCT with 32 patients who underwent single-level ACDF with PEEK cages filled with bone substitute. Likewise, the RCT by Niu et al. [13] resulted in a fusion rate of 100% in allograft filled PEEK cages at 12 months in 25 patients. Similarly, high rates of fusion were reported in cohort studies on filled PEEK cages. Two cohort studies reported high rates of 100% for filled PEEK cages fusion with follow-up period ranging from six to 12 months [14, 15]. A case series by Liao et al. [16] reported 100% fusion rate with a follow up of at least 12 months as well when using allograft filled PEEK cages. Subsidence rates with filled PEEK cages were reported to be 0% at six months by Cho et al. [14] and at 12 months by Niu et al. [13], and Liao et al. [16]. In recent case series by Godlweski et al. [17] on 100 patients following ACDF with PEEK cages filled with phosphocalcic hydroxyapatite, the subsidence rate was found to be 10.23% at 12 months follow-up.

Regarding empty PEEK cages, we found mixed results with radiographic outcomes with studies reporting lower fusion rates of 81.3–100% and higher subsidence rates of 0–48.3% compared to the filled PEEK cages. Results from a recent prospective multicenter study on 292 patients by Suess et al. [18] indicated poor performance of empty standalone PEEK cages. They found that these cages had slow radiographic fusion rates with an 83% complete fusion at 18 months, which translated into poor clinical outcomes. The authors recommended against the use of empty PEEK cages in ACDF, and filling cages with bone graft was advocated. In two retrospective studies by Klingler et al. [19] and Cabraja et al. [21], and a case series by Kim et al. [20], standalone cages had lower fusion of 65%, 81.3%, and 88.1% respectively. Furthermore, the subsidence rate ranged from 14.3 to 48.3% across these studies. The mean follow-up of the three studies ranged from 27.6 to 30 months. On the other hand, in a small series of 36 patients who underwent ACDF with empty self-locking PEEK cages, Kapetanakis et al. [22] reported complete fusion by 6 months without any subsidence at 12 months. However, this study is noncomparative with short duration and low number of subjects. Likewise, in two case series by Gok et al. [23] and Park et al. [24], empty PEEK cages had a high fusion rate of 92% in one-level ACDFs; however, they were associated with 33.8% (16 months follow-up) and 12% (12 months follow-up), respectively. Varying rates of fusion and subsidence were found in a large case series by Shiban et al. [25] on 265 patients following ACDF with empty PEEK cages. In this case series, the fusion rates were 85% for one-level fusions (127 patients), 95% for two-level fusions (125 patients), and 94% for three-level fusions (13 patients). The subsidence rate was 25% and 27% for one- and two-level fusions, whereas, three-level fusion has 15% subsidence.

### Clinical outcomes

Across the literature, several outcome measures were used to determine the results of standalone PEEK cages. The visual analogue scale (VAS) is used to measure the change in pain following treatment. The neck disability index (NDI) and the Japanese Orthopaedic Association (JOA) score for myelopathy are outcomes measures that are cervical spine disease specific. The Odom’s criteria are another outcome measure that is focused at the resolution of the preoperative symptoms following surgical treatment. Table 3 displays the outcome measures used the different studies in this narrative review.

**Table 3:** Summary of outcome measures in different studies on standalone PEEK cages

Author, year	VAS for neck pain	VAS for arm pain	Neck disability index	Odom’s criteria	JOA score	Modified JOA score
Niu et al., 2010				X		
Liao et al., 2008				X		
Zhou et al., 2011					X	
Cabraja et al., 2012	X	X	X	X		
Klingler et al., 2014	X	X	X			
Shiban et al., 2015	X	X				
Gok et al., 2016	X	X		X		
Park et al., 2016	X	X	X			X
Farrokhi et al., 2017				X		
Kapetanakis et al., 2017	X	X	X			
Ofluoglu et al., 2017	X	X		X		
Suess et al., 2017	X	X	X	X		
Godlewski et al, 2018	X	X	X			
Kim et al., 2018	X	X	X			X

*VAS* visual analogue scale, *JOA* Japanese Orthopaedic Association

#### Visual analogue scale

The VAS is continuous scale comprised of a horizontal or vertical line of 10 cm in length, where one end of the line denotes 0, indicating no pain, and the other end of the line denotes 10, indicating worst pain. A change by 2.5–4.1 in VAS for arm pain is considered beyond the minimal clinical important difference (MCID) and by 2.5–2.6 in VAS for neck pain [28, 29].

Two case-series reported the VAS in filled PEEK cages. Ofluoglu et al. [30] reported a 6.4 mean change in VAS for neck pain and 6.6 mean change in VAS for arm pain at 13 months following ACDF with ß-TCP filled PEEK cages. Godlewski et al. [17] found an improvement in the mean VAS by 2.84 at 12 months of follow-up with phosphocalcic hydroxyapatite filled PEEK cages.

Regarding empty PEEK cages, Suess et al. [18] reported in an RCT a mean change of 4 (interquartile range 3–6 if fused or 3–4 if not fused) following ACDF at 18 months. The rest of the studies on empty PEEK cages were case series. At mean of follow-up of 27.5 months, Kim et al. [20] found in a case series that ACDF with empty PEEK cages had a mean change of 1.98 for the VAS for neck pain for patients with subsidence and 2.83 for those without subsidence. Furthermore, the mean change for VAS for arm pain was 4.17 for patients with subsidence and 4.32 for those without subsidence. Moreover, Kapetanakis et al. [22] reported in a case series a mean change by 5.1 in VAS neck pain and 6.7 in VAS arm pain scores at 12 months of follow-up with empty PEEK cages. Additionally, Park et al. [24] found out that the change with empty PEEK cages resulted in a mean VAS change for pain by 2.1 points at a mean follow-up of 16 months of patients without subsidence and 21 months for subsidence patients.

#### Neck disability index

The NDI assesses the effect of neck pain on daily living and consists of ten items which are concerned with pain intensity, personal care, lifting objects, reading, headaches, concentration, performing work, driving, sleeping, and performing recreational activities [31]. Each item is scored from 0 to 5 with a total NDI score being 50 which reflects maximum disability. A change in the NDI by 7.5–10.5 points has been considered as a MCID [28, 32].

Only one case series reported NDI on filled PEEK cages. Godlewski et al. [17] reported in their mean change in NDI by 11.16 at 12 months. All patients in this series underwent ACDF with phosphocalcic hydroxylapatite–filled PEEK cages.

With regard to empty PEEK cages, Suess et al. [18] reported in their RCT an improvement in the NDI by a median of 22 and a range of 2–44 in 239 patients at 18 months of follow-up. In a retrospective cohort by Cabraja et al. [21], it was found that empty PEEK cages had similar functional outcomes compared with titanium cages at mean follow-up of 28.4 months (*p* = 0.940).

In addition, a case series by Kim et al. [20] found a change in mean change in NDI by 6.6 in empty PEEK cages who had subsidence and 7.8 in those who did not develop any subsidence at a mean follow-up of 27.6 months. Another case series by Kapetanakis et al. [22] reported a mean improvement in NDI following ACDF by empty cages from 57 pre-operatively to 24 points at 12 months, hence indicating a difference of 33 points. In a case series on 77 patients who underwent ACDF by empty PEEK cages, Park et al. [24] showed that patients had an improvement in NDI by 6.5–7.25 points in a follow-up period ranging from 13 to 85 months in 77 patients

#### Odom’s criteria

The Odom’s criteria were found in five studies in our narrative review. The Odom’s criteria [33] reflect the recovery of the resolution of cervical disc disease pre-operative findings following surgical treatment. The Odom’s criteria stratifies patients into four categories: Excellent if all pre-operative symptoms resolved and physical examination findings improved; Good if symptoms are mostly relieved with improvement or no change in physical examination findings; Fair if some relieve of symptoms is obtained with minimal improvement or no change in physical examination findings; Poor if no change or deterioration in symptoms and physical examination findings.

For filled PEEK cages, Niu et al. [13] reported in their RCT that 80% of patients who underwent ACDF with autograft filled PEEK cages to have excellent to good Odom’s scores at 12 months, and this was statistically insignificant compared to titanium cages (*p* = 0.6642). In another RCT by Farrokhi et al. [12], PEEK cages filled with bone substitute led to an excellent to good Odom’s score in 81.2% of patients at 12 months. This rate was less that of Acrylic cages which was 96.9% (*p* = 0.016). In the case series by Liao et al. [16], allograft filled PEEK cages in 19 patients achieved good to excellent Odom’s scores in 74% of patients. Moreover, Ofluoglu et al. [30] reported in their case series excellent to good Odom’s scores at 13 months in all patients who underwent ACDF by ß-TCP filled PEEK cages.

In unfilled PEEK cages, the RCT by Suess et al. [18] had 70.2% excellent to good Odom’s scores in their RCT at 18 months when empty PEEK cages were used. Moreover, the retrospective cohort by Cabraja et al. [21] found that empty cages had a 64.3% excellent-good Odom’s scores at a mean follow-up of 28.4 months in a retrospective cohort study. This finding was statistically indifferent from titanium cages (*p* = 0.229).

#### Japanese Orthopaedic Society (JOA) score

In our literature search, one study reported the JOA score and two studies reported the modified JOA score for myelopathy. The JOA score consists of six items which are scored separately [34]. The items reflect motor function in the upper and lower extremities; sensory function in trunk, upper and lower extremities; and bladder function. The lowest score possible is 0 indicating severest myelopathy, and the maximum score is 17. The modified JOA was introduced later by Benzel et al. [35] by excluding sensory function from the trunk and lower extremities, and it is scored with minimum score of 0 and a maximum score of 18. It has been reported that a change of two points in both the original JOA and the modified JOA is considered as the MCID [36, 37].

Zhou et al. [15] reported in a prospective cohort study that PEEK cages filled with autograft led to an increase in JOA by 6.5 points which translated into 93% JOA recovery rate at 12 months. Regarding empty PEEK cages, the case series by Park et al. [24] reported an increase of mJOA score from 0.92 to 1.34 points in 77 patients. Moreover, in another case series by Kim et al. [20], 68 patients who underwent ACDF with empty PEEK cages had an improvement in mJOA scores by 0.92 in patients who developed subsidence and 1.34 in patients without subsidence at a mean follow-up of 27.6 months.

### Complications

Post-operative complications are another important clinical outcome for ACDF with standalone PEEK cages. Cho et al. [14] reported the occurrence of pharyngitis in one patient out of 40 following fusion with filled PEEK cages. Fusion with filled cages have also been reported by Farrokhi et al. [12] to cause transient hoarseness of voice in two patients and led to two re-operations for disc disease at a lower level. Kapetanakis et al. [22] had 1 case of post-operative haematoma that required evacuation and 1 case of transient dysphagia following fusion with filled PEEK cages. Klingler et al. [19] reported 1 case of anterior empty PEEK cage dislocation that required revision surgery. Shiban et al. [25] had 16 revision surgery for adjacent segment disease and 4 reoperations for pseudarthrosis following fusion with empty peek cages. Lied et al. [26, 27] reported the occurrence of two neck haematomas that required evacuation and one neurological deterioration manifested as transient Horner’s syndrome and persistent left-sided myelopathy and reflex dystrophy. Table 4 displays the summary of complications in our review.

**Table 4:** Summary of post-operative complications following fusion by standalone PEEK cages

Complications	
18 Adjacent segment diseases requiring revision surgeries 4 Pseudarthrosis requiring revision surgeries 3 Post-operative haematoma requiring evacuation 2 Transient hoarseness of voice 2 Adjacent segment disease requiring revision surgeries 1 Pharyngitis 1 Transient dysphagia 1 Neurologic deterioration	

## Review strengths and limitations

This is a narrative review on the use of standalone PEEK cages in ACDF for degenerative cervical spine diseases. We have included 16 studies that were conducted from a period ranging from 2002 till 2018 with a total of 1088 patients. These studies have utilized different clinical outcomes and reported radiological outcomes in the form of subsidence and fusion rates.

However, this review is not without limitations. The mean follow-up period in these studies was at a maximum of 27.6 months in one study only. Moreover, the cumulative evidence at present is poor with only three RCTs, and the rest of studies were mostly case series and comparative cohort studies. Thus, one must be cautious when interpreting the results of this review due to the marked heterogeneity in the current evidence. Another limitation is the nature of this review being narrative; therefore, it does not systematically review the literature and does not employ meta-analytic comparisons for standalone PEEK cages against other modalities in ACDF.

## Conclusion

In this narrative review on the outcomes of standalone PEEK cages, several radiological and clinical outcomes were reviewed. Radiological outcome was described as fusion and subsidence rates following ACDF by PEEK cages. Filled PEEK cages was associated with high fusion and low subsidence rates compared with empty PEEK cages, as empty cages had fusion rates of 81.3–100% and subsidence rates of 0–48.3%. In terms with clinical outcomes, both filled and empty PEEK cages seemed to achieve change beyond the MCID for the NDI and VAS for neck and arm pain. However, filled PEEK cages had high percentages of excellent to good Odom’s scores and clinically important differences in JOA scores compared with empty PEEK cages. Regarding post-operative complications, the most encountered reason requiring revision was adjacent disc disease followed by pseudarthrosis and neck haematoma. In conclusion, standalone filled PEEK cages had better radiological outcomes and satisfactory clinical outcomes in terms of Odom’s score and JOA scores when compared with standalone unfilled PEEK cages. We advocate the use of filled PEEK cages which may translate into improved clinical outcomes. Further large randomized clinical trials are needed to compare the radiological outcomes and patient-reported outcomes between filled and unfilled standalone PEEK cages.
